# Magnesium-Based Absorbable Metal Screws for Intra-Articular Fracture Fixation

**DOI:** 10.1155/2016/9673174

**Published:** 2016-10-19

**Authors:** Roland Biber, Johannes Pauser, Markus Geßlein, Hermann Josef Bail

**Affiliations:** ^1^Nuremberg General Hospital, Paracelsus Medical University, Nuremberg, Germany; ^2^Department of Orthopaedics and Traumatology, Nuremberg General Hospital, Nuremberg, Germany

## Abstract

MAGNEZIX® (Syntellix AG, Hanover, Germany) is a biodegradable magnesium-based alloy (MgYREZr) which is currently used to manufacture bioabsorbable compression screws. To date, there are very few studies reporting on a limited number of elective foot surgeries using this innovative implant. This case report describes the application of this screw for osteochondral fracture fixation at the humeral capitulum next to a loose radial head prosthesis, which was revised at the same time. The clinical course was uneventful. Degradation of the magnesium alloy did not interfere with fracture healing. Showing an excellent clinical result and free range-of-motion, the contour of the implant was still visible in a one-year follow-up.

## 1. Introduction

Fixation of osteochondral fragments has to provide high stability, so that early mobilization and physiotherapy of the treated joint can be ensured. Current implants made of steel or titanium are able to meet these demands. However, in case the implant has to be removed due to complications, for example, the prominence of the head of a screw or if cartilage is lost postoperatively, a revision surgery becomes necessary. Biodegradable materials are an option to overcome this issue. This class of implants commonly consists of polymers, which lack adequate mechanical strength. Degradation of polymers is mostly facilitated by hydrolyses, only in some cases by enzymes. Hydrolyses, however, can result in an acid environment, favouring foreign body reactions and infections [1–3].

**Figure 1:**
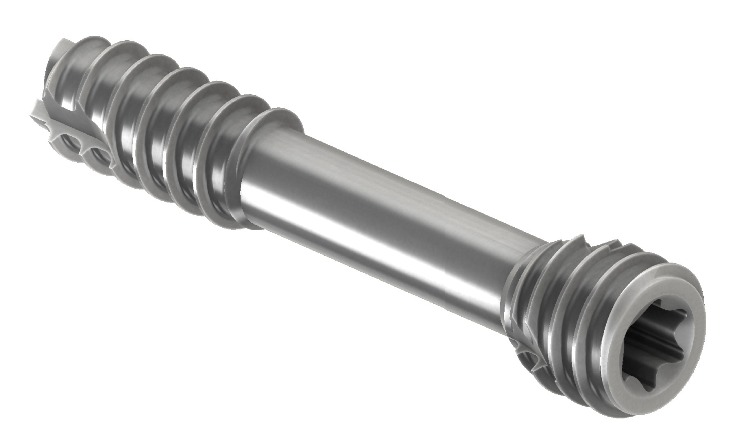
The compression screw MAGNEZIX CS resembles a cannulated conventional compression screw. However, it is made of a completely bioabsorbable magnesium alloy (MgYREZr).

Biodegradable magnesium-based implants are an innovative alternative. Here, several alloys have recently been studied in animal experiments [4–9]. In 2013, the MAGNEZIX screw (Syntellix AG, Hannover, Germany) was the first magnesium implant to be approved for application in humans worldwide. The MAGNEZIX 3.2 mm compression screw chemically consists of the magnesium-alloy MgYREZr (i.e., magnesium, yttrium, rare earth metal, and zirconium). It is available in a range of lengths from 10 mm to 40 mm (in 2 mm increments) (Figure 1).

Experimental studies on this material proved biocompatibility and osteoconductivity. Several studies on magnesium implants even revealed an osteogenic potential [1, 2, 4, 10–12]. There seems to be no potential for allergic effects [1]. The expected time until complete degradation is about one year as shown in an animal study [12].

Although more than 15.000 implants have been placed on the market, there are only limited publications about this innovation, mostly in the field of elective orthopaedic surgery. In particular, this experience is limited to foot surgery. One major study reports on fixations of 13 Chevron osteotomies with MAGNEZIX screws. Comparing the results to a control group fixed with titanium alloy screws, no disadvantages were identified [2]. Early results (1-year experience) have recently been published, reporting good clinical outcome after distal metatarsal osteotomies for hallux valgus in the short term and a high patient satisfaction [13]. Up to now, there are no clinical reports whatsoever on trauma applications.

## 2. Case Presentation

**Figure 2:**
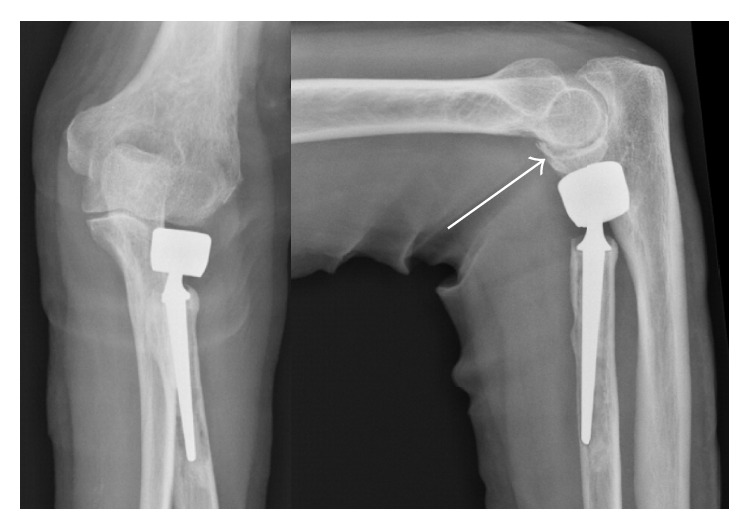
Surgery was indicated for both painful loosening of a radial head prosthesis and an osteochondral fracture of the capitulum humeri (white arrow).

We report the case of a 73-year-old female who suffered two falls within a short period. The first fall resulted in painful loosening of a radial head prosthesis. Waiting for the already scheduled operation, the second fall occurred, resulting in an additional fracture of the humeral capitulum (Figure 2). Via a lateral approach to the elbow, we performed revision of the radial head prosthesis, exchanging it for a cemented version. The large osteochondral fragment of the capitulum humeri was openly reduced, temporarily fixed by two Kirschner wires, and fixed with a MAGNEZIX compression screw (Ø 3.2 mm, length 32 mm).

Operative technique resembled that of conventional cannulated compression screws with placement of Ø 1.2 mm guide wire, drilling Ø 2.5 mm, countersink Ø 3.5 mm, and Ø 3.2 mm screw insertion; all steps are performed cannulating over guide wire.

Postoperative wound healing was uneventful. For mobilization, range-of-motion (ROM) was unrestricted, and the patient was advised with limited weight bearing (5 kg) for 6 weeks. A cast was applied for two weeks for wound healing protection only.

**Figure 3:**
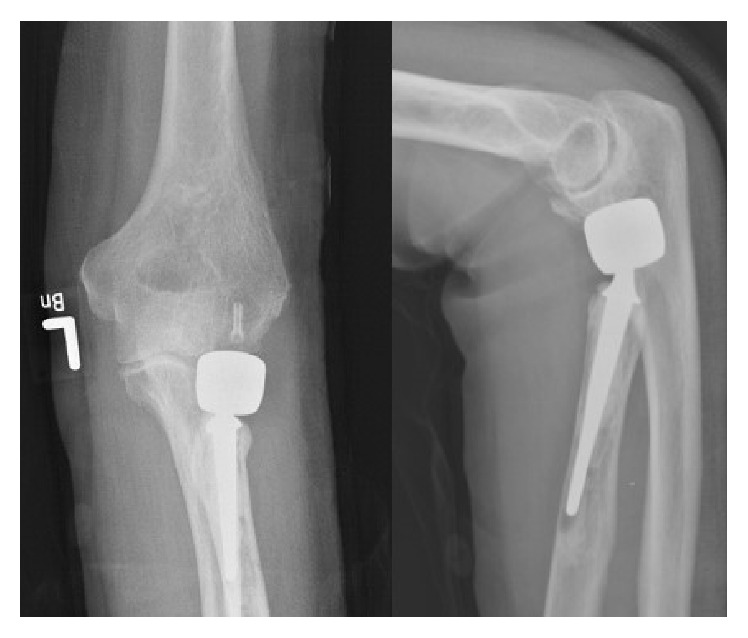
On postoperative X-ray, the MAGNEZIX CS can be identified as mildly radiopaque structure.

As the MgYREZr implant appears somewhat radiopaque, postoperatively X-rays allow correct implant placement to be checked without preventing the evaluation of the fracture area (Figure 3).

Further clinical course was uneventful. Physiotherapy was started and continued for 6 weeks, when unrestricted ROM was achieved (extension/flexion 0°-0°-120°). No adverse effects such as wound healing disturbance, swelling, or pain were noted.

**Figure 4:**
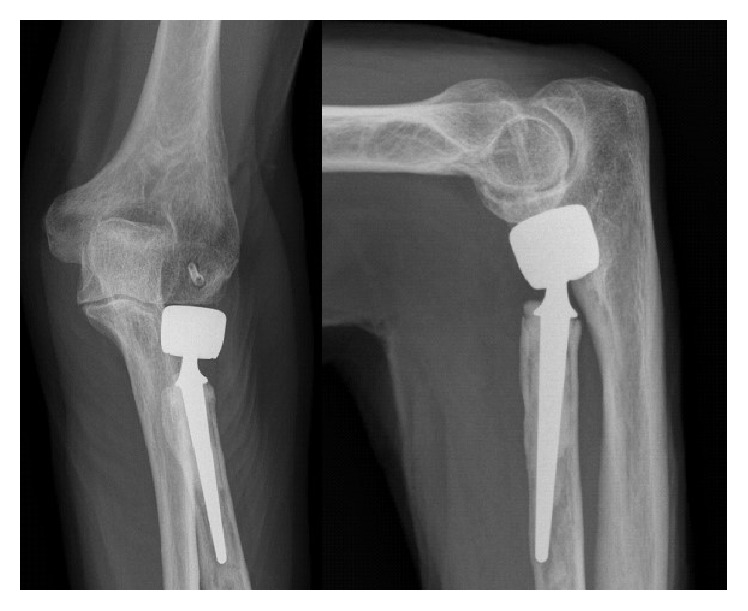
At one-year follow-up, the patient showed an excellent clinical result. The contour of the MAGNEZIX CS implant is still clearly visible.

At 1-year follow-up, the patient displayed an excellent clinical result, still with unrestricted ROM without pain, swelling, or other functional deficits. The contour of the implant was still visible on plain radiographs, and the surrounding bone and joint structures seemed radiographically undisturbed (Figure 4).

## 3. Discussion

Metal removal may be challenging especially for small, intra-articular implants inserted below the surface of the cartilage. Considerable field damage may occur; thus, the decision for implant removal is taken with caution nowadays. Earlier publications suggest that steel or titanium alloy screws should be routinely removed, if used for fixation of osteochondral fragments [14, 15]. This suggestion changed over time to remove metal screws only, if complications occur. To further reduce the rate of revision surgery, biodegradable implants have been introduced [16]. The rate of metal implant removal in osteochondral fractures, even in knee joints after patella dislocation, is not exactly known [16, 17]. Aydoğmuş et al. reported in their recent publication a case of patellofemoral implant friction after the refixation of an osteochondral fragment with two headless metal compression screws [18]. For the rare procedure of fixation of osteochondral fragments of the capitellum, only case reports have been identified [19, 20].

Usage of biodegradable, nevertheless stable metal screws would represent a remarkable advantage regarding this issue. Theoretical applications include all kinds of screw fixation in small bones as well as fixation of small fragments including osteochondral flakes. The MAGNEZIX screw is approved for these indications (CE mark, HSA approval (Singapore)). The manufacturer explicitly recommends this implant for intra- and extra-articular fractures, nonunions, bone fusion, bunionectomies, and osteotomies [21].

Up to now, there is limited experience about the clinical application of the MAGNEZIX CS. Reports currently focus on elective foot surgery such as Chevron-type osteotomies of the first metatarsal bone [2, 13]. Our report now expands the application into the field of trauma surgery. After fixation of an intra-articular elbow fracture with a MAGNEZIX CS 3.2, we observed uneventful healing both clinically and radiologically. Radiologic follow-up did not detect any evidence for interference of any implant degradation products with fracture healing.

Our finding of an uneventful consolidation of the osteochondral fracture of the elbow after MAGNEZIX screw fixation is consistent with the studies of Windhagen et al., who also reported normal bone consolidation without any radiographic abnormalities around their Chevron osteotomies [2]. Degradation of magnesium alloys is known to produce hydrogen, which can form cavities within the tissue [10, 22]. Animal experiments with 1-year follow-up, however, indicated no associated bone loss [12]. In our case, no radiolucent zones were detected in the area of the implant. However, a computed tomography (CT) was not performed due to the unnecessary exposure of the patient to radiation.

Operative technique and handling of the MAGNEZIX CS were completely equivalent to conventional metal screws made of titanium. Although magnesium alloys have generally lower Young's Modulus than titanium alloys [6, 10, 12], applied torques and intraoperative stability appeared comparable to titanium implants. Degradation studies showed an implant mass reduction of less than 10% during the first six weeks, with the pull-out forces even increasing after four weeks [5]. In animal experiments, complete degradation of MgYREZr implants takes about one year [12]. Our finding of a radiographically visible screw after one year should not necessarily be interpreted as a fully intact metallic screw. Waizy et al. have shown that after 12 months the screw has turned to an apatite formation possessing a high density [12]. MRI scans after 36 months revealed that the former implants site becomes saturated with bone tissue (partially cancellous or cortical) following the degradation of the implant [23]. Therefore, it can be speculated that the now visible contour of the implant may resemble bone tissue.

## 4. Conclusion

The MAGNEZIX CS is a fully degradable implant made of magnesium alloy [MgYREZr]. Reports on clinical applications are limited to a relatively small number of Chevron osteotomies in the past. This is the first report on a trauma application, where the implant was used for an intra-articular fracture fixation in the elbow. The clinical and radiological course was uneventful. On one-year follow-up, the contour of the implant was still visible on plain radiographs. Further clinical reports are needed in order to describe clinical and radiological outcomes of this innovative implant.

## References

[1] Luthringer B. J. C., Feyerabend F., Willumeit-Römer R. (2014). Magnesium-based implants: a mini-review. *Magnesium Research*.

[2] Windhagen H., Radtke K., Weizbauer A. (2013). Biodegradable magnesium-based screw clinically equivalent to titanium screw in hallux valgus surgery: Short term results of the first prospective, randomized, controlled clinical pilot study. *BioMedical Engineering Online*.

[3] Seitz J.-M., Durisin M., Goldman J., Drelich J. W. (2015). Recent advances in biodegradable metals for medical sutures: a critical review. *Advanced Healthcare Materials*.

[4] Bondarenko A., Angrisani N., Meyer-Lindenberg A., Seitz J. M., Waizy H., Reifenrath J. (2014). Magnesium-based bone implants: immunohistochemical analysis of peri-implant osteogenesis by evaluation of osteopontin and osteocalcin expression. *Journal of Biomedical Materials Research—Part A*.

[5] Erdmann N., Angrisani N., Reifenrath J. (2011). Biomechanical testing and degradation analysis of MgCa0.8 alloy screws: a comparative in vivo study in rabbits. *Acta Biomaterialia*.

[6] Erdmann N., Bondarenko A., Hewicker-Trautwein M. (2010). Evaluation of the soft tissue biocompatibility of MgCa0.8 and surgical steel 316 L in vivo: a comparative study in rabbits. *BioMedical Engineering Online*.

[7] Reifenrath J., Angrisani N., Erdmann N. (2013). Degrading magnesium screws ZEK100: biomechanical testing, degradation analysis and soft-tissue biocompatibility in a rabbit model. *Biomedical Materials*.

[8] Heublein B., Rohde R., Kaese V., Niemeyer M., Hartung W., Haverich A. (2003). Biocorrosion of magnesium alloys: a new principle in cardiovascular implant technology?. *Heart*.

[9] Aghion E., Levy G., Ovadia S. (2012). In vivo behavior of biodegradable Mg-Nd-Y-Zr-Ca alloy. *Journal of Materials Science: Materials in Medicine*.

[10] Staiger M. P., Pietak A. M., Huadmai J., Dias G. (2006). Magnesium and its alloys as orthopedic biomaterials: a review. *Biomaterials*.

[11] Zhang E., Xu L., Yu G., Pan F., Yang K. (2009). In vivo evaluation of biodegradable magnesium alloy bone implant in the first 6 months implantation. *Journal of Biomedical Materials Research Part A*.

[12] Waizy H., Diekmann J., Weizbauer A. (2014). In vivo study of a biodegradable orthopedic screw (MgYREZr-alloy) in a rabbit model for up to 12 months. *Journal of Biomaterials Applications*.

[13] Plaass C., Ettinger S., Sonnow L. (2016). Early results using a biodegradable magnesium screw for modified chevron osteotomies. *Journal of Orthopaedic Research*.

[14] Matsusue Y., Nakamura T., Suzuki S., Iwasaki R. (1996). Biodegradable pin fixation of osteochondral fragments of the knee. *Clinical Orthopaedics and Related Research*.

[15] Aichroth P. (1971). Osteochondritis dissecans of the knee. A clinical survey. *The Journal of Bone & Joint Surgery—British Volume*.

[16] Walsh S. J., Boyle M. J., Morganti V. (2008). Large osteochondral fractures of the lateral femoral condyle in the adolescent: outcome of bioabsorbable pin fixation. *The Journal of Bone & Joint Surgery—American Volume*.

[17] Kühle J., Südkamp N. P., Niemeyer P. (2015). Osteochondral fractures at the knee joint. *Unfallchirurg*.

[18] Aydoğmuş S., Duymuş T. M., Keçeci T. (2016). An unexpected complication after headless compression screw fixation of an osteochondral fracture of patella. *Case Reports in Orthopedics*.

[19] Silveri C. P., Corso S. J., Roofeh J. (1994). Herbert screw fixation of a capitellum fracture. A case report and review. *Clinical Orthopaedics and Related Research*.

[20] Sodl J. F., Ricchetti E. T., Huffman G. R. (2008). Acute osteochondral shear fracture of the capitellum in a twelve-year-old patient: a case report. *Journal of Bone and Joint Surgery—Series A*.

[21] Seitz J.-M., Lucas A., Kirschner M. (2016). Magnesium-based compression screws: a novelty in the clinical use of implants. *JOM*.

[22] Song G., Atrens A. (2003). Understanding magnesium corrosion—a framework for improved alloy performance. *Advanced Engineering Materials*.

[23] Modrejewski C., Plaass C., Ettinger S. Degradation behaviour of Magnesium alloy screws after distal metatarsal osteotomies in MRI.

